# Association of *P2Y12* Gene Promoter DNA Methylation with the Risk of Clopidogrel Resistance in Coronary Artery Disease Patients

**DOI:** 10.1155/2014/450814

**Published:** 2014-03-18

**Authors:** Jia Su, Xiaojing Li, Qinglin Yu, Yahui Liu, Yaqing Wang, Haojun Song, Hanbin Cui, Weiping Du, Xiaohong Fei, Junsong Liu, Shaoyi Lin, Jian Wang, Wenyuan Zheng, Jinyan Zhong, Lulu Zhang, Maoqing Tong, Jin Xu, Xiaomin Chen

**Affiliations:** ^1^Department of Cardiology, The Affiliated Ningbo No. 1 Hospital, School of Medicine, Ningbo University, Ningbo, Zhejiang 315010, China; ^2^Department of Traditional Chinese Internal Medicine, The Affiliated Ningbo No. 1 Hospital, School of Medicine, Ningbo University, Ningbo, Zhejiang, China; ^3^The Key Laboratory of Molecular Medicine, The Affiliated Ningbo No. 1 Hospital, School of Medicine, Ningbo University, Ningbo, Zhejiang, China; ^4^Department of Gastroenterology, The Affiliated Ningbo No. 1 Hospital, School of Medicine, Ningbo University, Ningbo, Zhejiang, China; ^5^Institute of Preventative Medicine, School of Medicine, Ningbo University, Ningbo, Zhejiang, China

## Abstract

*Background*. Clopidogrel inhibits the ADP receptor *P2Y12* to keep down the platelet aggregation. The goal of our study is to investigate the contribution of *P2Y12* promoter DNA methylation to the risk of clopidogrel resistance (CR). *Methods*. The platelet functions were measured by the VerifyNow *P2Y12* assay. Applying the bisulfite pyrosequencing technology, DNA methylation levels of two CpG dinucleotides on *P2Y12* promoter were tested among 49 CR cases and 57 non-CR controls. We also investigated the association among *P2Y12* DNA methylation, various biochemical characteristics, and CR. *Result*. Lower methylation of two CpGs indicated the poorer clopidogrel response (CpG1, *P* = 0.009; CpG2, *P* = 0.022) in alcohol abusing status. Meanwhile CpG1 methylation was inversely correlated with CR in smoking patients (*P* = 0.026) and in subgroup of Albumin < 35 (*P* = 0.002). We observed that the level of DNA methylation might be affected by some clinical markers, such as TBIL, LEVF, Albumin, AST. The results also showed that the quantity of stent, fasting blood-glucose, and lower HbAC1 were the predictors of CR. *Conclusions*. The evidence from our study indicates that *P2Y12* methylation may bring new hints to elaborate the pathogenesis of CR.

## 1. Introduction

ADP activates platelets through two G protein-coupled receptors:* P2Y1* and* P2Y12*, and the activation of* P2Y12* leads to sustained platelet aggregation [1]. Clopidogrel inhibits the purinergic ADP receptor* P2Y12*, keeps down the adenosine-diphosphate-induced platelet aggregation, and then prevents the cardiovascular risks in coronary atherosclerotic heart disease (CAD) patients who have been undergoing percutaneous coronary intervention (PCI) [2]. However, the pharmacodynamic response to clopidogrel varies greatly [3], and patients with lesser degrees of platelet inhibition are more likely to suffer ischemic events [4, 5].

Clopidogrel resistance (CR) has been used to reflect the failure of clopidogrel to achieve its effect of antiplatelet aggregation. The underlying mechanism remains unclear. The extrinsic or intrinsic factors may contribute to the variability of platelet activity, including the role of genetic polymorphisms of transporters and enzymes participating in clopidogrel absorption and metabolic transformation and nongenetic causes, such as drug-drug interactions, comorbidities, and age [6]. Genetic factors, specifically the expression of the gene* P2Y12, *may play a great role in individual susceptibility to the clopidogrel [7].

DNA methylation is a reliable epigenetic marker and specifically occurs in the context of cytosine-phosphate-guanine (CpG) dinucleotide [8]. Vertebrate CpG islands (CGIs), which are briefly interspersed CpG-rich DNA sequences, are not predominantly methylated in or near the promoters of mammalian genes [9]. CGI hypermethylation is linked to transcriptional silencing of gene expression [10] and thus regulates the level of protein.

The evidence of the association between DNA methylation and the risk of clopidogrel resistance was scarce. Since multiple single-nucleotide polymorphisms (SNPs) associated with poor activity to clopidogrel [11] did not appear to directly affect P2Y12 expression, the altered expression of* P2Y12* was likely to be modulated by methylations of promoters or other epigenetic changes. Thus, we attempted to assess whether the DNA methylations of selected CpG islands in* P2Y12* gene promoter were involved in clopidogrel resistance. Due to the little knowledge so far, this study may help to explore a novel aspect in understanding the mechanism of clopidogrel resistance.

## 2. Methods

### 2.1. Study Population

One hundred and six patients with CAD were collected from the Ningbo No. 1 Hospital. All of them were Han Chinese originated from Ningbo city in Eastern China. The inclusion criteria were as follows. (1) Patients for PCI: with drug-eluting stent, PCI was carried out according to current standard guidelines (ACC/AHA guidelines) through the radial route. (2) All the individuals were administrated with a loading dose of aspirin (300 mg) as well as clopidogrel (300 mg) before PCI. followed by 75 mg of clopidogrel and 100 mg of aspirin daily. (3) Patients of age ≥18 years and ≤80 years were included. The exclusion criteria were as follows: (1) the administration of concomitant glycoprotein IIb/IIIa inhibitor administration, (2) recent or chronic clopidogrel treatment, (3) sudden death, (4) history of bleeding diathesis, (5) haematocrit < 35% or >50%, and (6) total platelet count <150 000 *μ*L or >500 000 *μ*L.

Written informed consent was obtained from all the subjects. The study was approved by the Ethics Committee of Ningbo No. 1 Hospital and conformed with the principles outlined in the Declaration of Helsinki.

### 2.2. Collection of Samples and Clinical Data

Blood samples were obtained overnight fast from the antecubital vein. The serologic markers, such as the concentrations of TG, LDL, ALT, AST, and uric acid in plasma, were measured by the IFCC reference measurement systems. All the tests applied the standard procedures recommended by the manufacturers and then the data were collected and entered into a central database.

### 2.3. Platelet Function Measurements

As there may be no significant changes of platelet reactivity from days 3 to 5 in AMI patients undergoing PCI [4], we measured the patients' platelet reactivity at one week to one month after PCI. Blood samples were collected using the double-syringe technique and the first 2 to 4 mL free flowing blood was discarded to avoid spontaneous platelet activation. The platelet functions were measured by the VerifyNow* P2Y12* assay (Accumetrics Inc., San Diego, CA).

The VerifyNow* P2Y12* Assay is a whole blood, point-of-care system that has been developed to evaluate the responsiveness to* P2Y12* antagonists [12]. Blood is drawn into a Greiner Bio-One 3.2% citrate Vacuette tube (Greiner Bio-One, Kremsmünster, Austria). The assay instrument contains fibrinogen-coated polystyrene beads, 20 nmol/L ADP, and 22 nmol/L PGE1. Surveyed by an optical signal, the result was expressed as* P2Y12* reaction units (PRU). And a residual platelet reactivity (RPR) cutoff value ≥240 reaction units indicated the existence of clopidogrel resistance [13].

### 2.4. DNA Methylation Assay

Human genomic DNA was extracted from leucocytes of peripheral blood samples with a commercially available kit (QIAamp DNA Blood Mini Kit, Qiagen, Hilden, Germany). DNA concentrations were quantified by the ultramicronucleic acid ultraviolet tester (NANODROP 1000, Wilmington, USA), and all of them were more than 500 ng/*μ*L.

Bisulfite pyrosequencing technology was applied to determine the methylation levels of 2 CpG dinucleotides on the fragment of* P2Y12* gene promoter, which combines with sodium bisulfite DNA conversion chemistry (EpiTech Bisulfite Kits; Qiagen), polymerase chain reaction (PCR) amplification (Pyromark PCR Kit; Qiagen), and sequencing by synthesis assay (Pyromark Gold Q24 Reagents; Qiagen) of the target sequence. We selected PyroMark Assay Design software for planning PCR primers. Sequences of the PCR and pyrosequencing primers were described in Table 1.

### 2.5. Statistical Analysis

All data for continuous variables were described as means ± standard deviation and skewed variables as the median with interquartile range (IQR). A series of statistical analyses were performed to investigate the association among* P2Y12* DNA methylation, various biochemical characteristics, and clopidogrel resistance.

For analysis of the association between categorical variables, we used either Pearson's chi-square or Fisher's exact test when appropriate. *t*-test or Wilcoxon rank sum test for unpaired samples was applied to compare any continuous variables. The multiple linear regression was implemented to determine the correlation between the promoter DNA methylation of* P2Y12* gene and metabolic factors. The interaction of P2Y12 methylation and confounding variables was tested by logistic regression.

A two-tailed *P* value less than 0.05 was considered to indicate statistical significance. All the statistical analyses above were carried out by PASW Statistics 18.0 software (SPSS, Inc., Somers, NY, USA).

## 3. Results

From October 2012 to October 2013, a total of 106 CAD patients who met the inclusion criteria were recruited in the current association study. Among them, by the VerifyNow* P2Y12* assay, the PRU of 49 patients was more than 240, and they are defined as having poor reactivity to clopidogrel or clopidogrel resistance. As shown in Table 2, the baseline characteristics of the cases and controls in our study were summarized. The clinical characteristics were matching except the Albumin. One with clopidogrel resistance was more likely to have lower Albumin (cases versus controls: 37.73 ± 5.03 versus 39.95 ± 5.02, *P* = 0.026).

**Figure 1:**
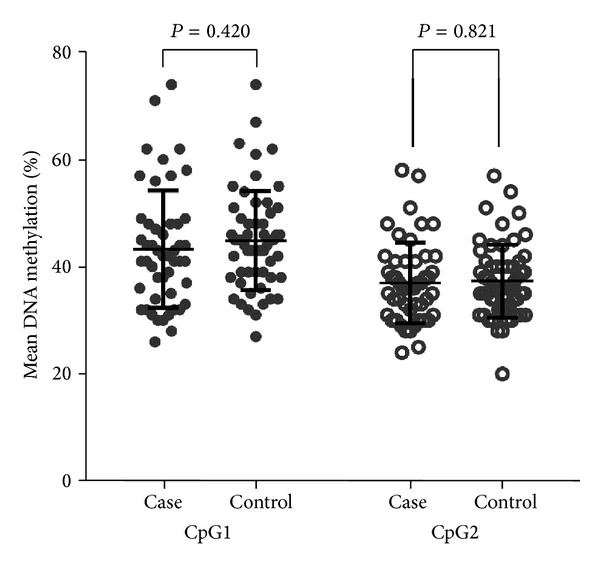
Comparison of* P2Y12 *methylation levels between cases and controls.* P2Y12* CpG1 (cases versus controls (%): 43.31 ± 10.96 versus 44.89 ± 9.26, *P* = 0.420) and* P2Y12* CpG2 (cases versus controls (%): 37.00 ± 7.52 versus 37.32 ± 6.82, *P* = 0.821).

In this study, we selected a fragment (GRCh37.p13:151103600-151101600) containing 2 CpG dinucleotides. Through bisulfite pyrosequencing assay, we explored the association of* P2Y12* gene promoter DNA methylation with clopidogrel resistance. Meanwhile, as shown in Figure 1 and Table 2, our results showed that* P2Y12* CpG1 methylation levels were not significantly associated with clopidogrel resistance (cases versus controls (%): 43.31 ± 10.96 versus 44.89 ± 9.26, *P* = 0.420), so as* P2Y12* CpG2 (cases versus controls (%): 37.00 ± 7.52 versus 37.32 ± 6.82, *P* = 0.821) too.

We performed a breakdown analysis by clinical characteristics to evaluate whether the methylation levels of* P2Y12* gene promoter (including CpG1 and CpG2) were related to clopidogrel poor response. It became clear to us that a significant association existed in the subgroup of Albumin < 35, current smoking, and alcohol abuse in the present study (Tables 3 to 5). We discovered that if patients were tippling, lower CpG1 (Table 3, cases versus controls (%): 36.67 ± 7.25 versus 46.70 ± 7.42, *P* = 0.009) and CpG2 (Table 3, cases versus controls (%): 31.89 ± 5.18 versus 38.70 ± 6.48, *P* = 0.022) methylation levels both indicated the poorer clopidogrel response. Moreover, CpG1 methylation was inversely correlated with CR in smoking patients (Table 4, cases versus controls (%): 38.53 ± 6.87 versus 44.90 ± 10.03, *P* = 0.026). Similarly, lower levels of* P2Y12* CpG1 methylation were notably interrelated with increased risk of CR in subgroup of Albumin < 35 (Table 5, cases versus controls (%): 34.14 ± 7.24 versus 44.70 ± 4.45, *P* = 0.002). For the above two effects, no significant interactions were identified in* P2Y12* CpG2, and we did not find any other significant association in the rest of the subgroups.

We applied the method of multiple linear regression to explore the effect on DNA methylation from clinical factors. And we observed that the level of DNA methylation might be affected by some clinical marks (Table 6), such as TBIL, LEVF, Albumin, and AST in* P2Y12* CpG 1 with alcohol abuse (*F* = 9.302, *P*_*F* value_ = 0.001, *R* square = 0.724); LEVF, Albumin, and AST in P2Y12 CpG 2 with alcohol abuse (*F* = 10.033, *P*_*F* value_ = 0.001, *R* square = 0.667); LEVF and TBIL in P2Y12 CpG 1 with current smoking (*F* = 6.193, *P*_*F* value_ = 0.005, *R* square = 0.251); and LEVF, CRP, BUN, and Triglycerides in* P2Y12* CpG 1 with Albumin < 35 (*F* = 14.345, *P*_*F* value_ < 0.001, *R* square = 0.840).

Considering the influence of confounding variables, we carried out logistic regression analysis with nongenetic and genetic factors. The result showed that the quantity of stent and fasting blood-glucose were associated with CR, while the HbAC1 was inversely correlated with it (*P* < 0.05, Table 7). However, if we applied the logistic regression analysis with these in the above subgroups, it was indicated that the above nongenetic and genetic factors were no longer correlated with the poorer clopidogrel response (*P* > 0.05, Tables 8, 9, and 10).

## 4. Discussion

Since various trails had been keen on the single-nucleotide polymorphisms, some had averted their sight to epigenetics, such as miRNA, DNA methylation. Recently, we have found that aberrant methylation is interpreted to take part in the occurrence and development of diseases including colorectal cancer [14, 15], breast cancer [16, 17], coronary artery disease [18], and schizophrenia [19]. For clopidogrel resistance, numerous studies have investigated the underlying mechanism. The single-nucleotide polymorphisms (SNPs) within* P2Y12* gene have been extensively studied and some research has shown that the H2 haplotype (the* P2Y12* T744C polymorphism) influences the platelet aggregation [11]. Though more studies have paid attention to the epigenetic mechanisms in cardiovascular disease, especially clopidogrel resistance, for example, miRNA [20], there is little research focusing on the relationship between DNA methylation and the risk of clopidogrel resistance.

In this study, the relationship between* P2Y12* gene promoter DNA methylation and the risk of clopidogrel resistance in CAD patients was investigated. As far as we know, it was the first study on the topic of DNA methylation and clopidogrel resistance. Unfortunately, no correlation in selected CpG islands was detected. However, we found that a significant association existed in the subgroup of Albumin <35, current smoking, and alcohol abuse. The lower the methylation levels of CpG1 in the above subgroup, the higher the platelet activity after standardized antiplatelet treatment, similar to the methylation levels of CpG2 in alcohol abuse group too.

Although no public evidence had shown the value of Albumin and alcohol abuse would influence the clopidogrel responding, a new meta-analysis reported that the clinical benefit of clopidogrel treatment in reducing cardiovascular events (including death, myocardial infarction, and stroke) was discovered primarily in smokers, with little benefit in nonsmokers [21]. Thus, we need to give careful consideration to these factors. However, not every study demonstrated the significant association, especially the smoking status receiving clopidogrel. The basic research of their in-depth mechanism, which did not have a definite conclusion, should continue in order to give us ongoing insights.

Additionally, we have to observe the influence of confounding variables on methylation. In our study, some clinical factors (e.g., TBIL, LEVF, Albumin, AST CRP, BUN, and Triglycerides) may influence the level of methylation in a particular population, though the *R* square in model of* P2Y12* CpG 1 with current smoking was lower which indicates a lack of reliability. It was similar to other research that the concentration of AST was inversely correlated with* ADD1* CpG2-5 methylation levels in female nonessential hypertension [22], and the level of methylation in gene-specific DNA is associated with serum levels of C-reactive protein [23] as well as the levels of Triglycerides and fatty acids in abdominal adipose tissue [24]. However, of all research above including our study, the sample was limited. Additional and larger studies would enhance reliability.

Furthermore, we should focus on the gene-environment interaction. Increasing evidence manifested that environmental and lifestyle factors could influence epigenetic mechanisms, though few were involved in cardiology. For instance, one recent study, which evaluated global DNA methylation from buccal cells of children exposed to smoking, demonstrated hypomethylation of LINE-1 repetitive elements [25]. Another one pointed out that the acute exposure to ethanol induced high methylation level of specific cell cycle genes in monolayer cultures of neural stem cells [26]. In our study, beside the relationship between* P2Y12* gene methylation and the risk of clopidogrel resistance, we could not ignore the gene-environment interaction. Due to such a high interference factor, we wish to apply the multifactor dimensionality reduction (MDR), stratified analysis, and crossover analysis to identify and characterize the effect among the* P2Y12* methylation and the nongenetic factors [27]. However, it is too difficult to stratify the level of methylation in accepted standards.

Meanwhile, a large quantity of research indicates that some other extrinsic factors, comorbidities, for instance, might also contribute to clopidogrel resistance. We used the logistic regression analysis with confounding factors. In the total population, the quantity of stent, fasting blood-glucose, and lower HbAC1 were the predictors of CR. However, if we applied the logistic regression analysis in subgroups (Albumin < 35, current smoking, and alcohol abuse), all of them, including genetic and nongenetic causes, association were not found. Comorbidities, such as diabetes mellitus (including the type of diabetes [28] and glycemic control [29]), chronic renal diseases [30] or different renal function grades [31], obesity [31] or elevated body mass index (BMI), heart failure [13], and inflammatory state [32], might be the predictor of poor clopidogrel response. Some of the evidence above was similar to ours. Nevertheless, we observed that lower HbAC1 was the predictor of CR. It was opposite to recent studies of evidence-based medicine [33, 34]. This may be due to the limited samples and the lack of more consistent stratification. Additionally, a larger sample set of the population and more meticulous stratification standards would help us obtain a comprehensive understanding. Furthermore, as coexisting polymorphisms in different genes might lead to persistent platelet activation while on clopidogrel [35], sophisticated gene-gene interaction of DNA methylation, such as the DNA methylation in* ABCB1 *or* CYP2C19 *with* P2Y12*, also needs to be considered carefully. If future research is possible, the observations will likely bring new information to elaborate the pathogenesis of clopidogrel resistance.

Although considerable efforts have been made, there are some limitations inherent in our study. Firstly, the sample size is relatively small. Future investigation with larger samples can be arranged for further assessment. Secondly, for the whole promoter of* P2Y12* gene, we only selected one fragment of the CGI, but there might be other fragments related to clopidogrel resistance. Thirdly,* P2Y12* gene methylation was measured in DNA from the leucocyte in peripheral blood which contained granulocytes, lymphocytes, and so on. Fourthly, a possibility of unknown confounding factors might exist and affect the expression of* P2Y12* gene methylation. The exact interactions among them remain to be explored in future studies.

In summary, our study indicates that lower* P2Y12* gene promoter DNA methylation increases the risk of clopidogrel resistance in the patients with Albumin ≤ 35, current smoking, or alcohol abuse. Meanwhile some extrinsic factors might be correlated with DNA methylation, and the quantity of stent, fasting blood-glucose, and lower HbAC1 might be the predictors of clopidogrel resistance. Thus, we would aim for additional, larger studies and a more advanced empirical approach along with standardized stratification to validate our findings in further cases.

## Figures and Tables

**Table 1:** Primers for *P2Y12* gene CpG island loci analysis.

Group	DNA sequence
Forward primer	5′-AGGAATTTATAGGTTTATAAGTGATGATAT-3′
Reverse primer	5′-Biotin-CCTTCATTATAATTTCTATCCCACTTCTCA-3′
Sequencing primer	5′-GGTTTATAAGTGATGATATTATATG-3′

**Table 2:** Characteristics comparison between CR and non-CR.

	Cases (*n* = 49)	Controls (*n* = 57)	*P* value
Male gender, *n* (%)	35 (71.43)	46 (80.70)	0.262
Hypertension, *n* (%)	36 (73.47)	36 (63.16)	0.257
Diabetes mellitus, *n* (%)	10 (20.41)	13 (22.81)	0.765
Dyslipidemia, *n* (%)	20 (40.82)	26 (45.61)	0.619
Current smoking, *n* (%)	19 (38.78)	21 (36.84)	0.838
Alcohol abuse	9 (18.37)	10 (17.54)	0.912
Age, y	65.20 ± 11.50	61.74 ± 11.11	0.120
BMI, kg/m^2^,	23.84 ± 2.86	24.05 ± 2.74	0.695
Number of stents per patient	1.55 ± 0.98	1.28 ± 0.77	0.116
Left ventricular ejection fraction, %	59.04 ± 10.07	61.75 ± 7.45	0.115
Total cholesterol, mg/dL	4.57 ± 1.24	4.80 ± 1.36	0.382
Triglycerides, mg/dL	1.70 ± 1.02	1.73 ± 1.30	0.907
HDL cholesterol, mg/dL	0.95 ± 0.23	0.98 ± 0.31	0.608
LDL cholesterol, mg/dL	2.81 ± 1.06	2.96 ± 0.99	0.459
GLU, mmol/L	6.12 ± 2.42	5.69 ± 1.75	0.284
HbA1c, %	6.23 ± 1.31	6.22 ± 1.42	0.972
ALT, umol/L	39.67 ± 32.75	38.95 ± 31.56	0.908
AST, umol/L	125.27 ± 183.75	126.30 ± 183.16	0.977
TBIL, umol/L	15.54 ± 9.21	14.35 ± 7.42	0.464
Albumin (A), g/L	37.73 ± 5.03	39.95 ± 5.02	**0.026**
BUN, mmol/L	5.83 ± 2.10	5.61 ± 2.30	0.618
CREA, mmol/L	72.53 ± 20.33	71.56 ± 20.06	0.807
UA, umol/L	342.71 ± 102.48	321.26 ± 84.86	0.241
hsCRP, mg/L	10.13 ± 17.91	12.00 ± 21.95	0.636
PLT, ∗10^9^/L	191.22 ± 61.93	208.35 ± 85.68	0.248
MPV, fL	8.47 ± 1.40	8.03 ± 0.89	0.058
PCT, %	0.16 ± 0.04	0.17 ± 0.05	0.375
PDW, %	16.37 ± 0.63	16.32 ± 0.52	0.664
*P2Y12* CpG1, (%)	43.31 ± 10.96	44.89 ± 9.26	0.420
*P2Y12* CpG2, (%)	37.00 ± 7.52	37.32 ± 6.82	0.821

**Table 3:** Comparison of *P2Y12* methylation levels between cases and controls in subgroup of alcohol abuse or not.

	Alcohol abuse			No alcohol abuse		
	Cases (*n* = 9) Mean ± s.e.	Controls (*n* = 10) Mean ± s.e.	*P* value	Cases (*n* = 40) Mean ± s.e.	Controls (*n* = 47) Mean ± s.e.	*P* value
*P2Y12* CpG1, (%)	36.67 ± 7.25	46.70 ± 7.42	**0.009**	44.80 ± 11.17	44.51 ± 9.63	0.897
*P2Y12* CpG2, (%)	31.89 ± 5.18	38.70 ± 6.48	**0.022**	38.15 ± 7.53	37.02 ± 6.93	0.469

**Table 4:** Comparison of *P2Y12* methylation levels between cases and controls in subgroup of current smoking or not.

	Current smoking			No current smoking		
	Cases (*n* = 19) Mean ± s.e.	Controls (*n* = 21) Mean ± s.e.	*P* value	Cases (*n* = 30) Mean ± s.e.	Controls (*n* = 36) Mean ± s.e.	*P* value
*P2Y12* CpG1, (%)	38.53 ± 6.87	44.90 ± 10.03	**0.026**	46.33 ± 12.05	44.94 ± 9.04	0.598
*P2Y12* CpG2, (%)	34.00 ± 5.30	37.33 ± 7.35	0.112	38.90 ± 8.15	37.29 ± 6.70	0.384

**Table 5:** Comparison of *P2Y12* methylation levels between cases and controls in subgroup of Albumin <35 or not.

	Albumin < 35			Albumin ≥ 35		
	Cases (*n* = 7) Mean ± s.e.	Controls (*n* = 10) Mean ± s.e.	*P* value	Cases (*n* = 42) Mean ± s.e.	Controls (*n* = 47) Mean ± s.e.	*P* value
*P2Y12* CpG1, (%)	34.14 ± 7.24	44.70 ± 4.45	**0.002**	44.83 ± 10.78	44.94 ± 10.02	0.963
*P2Y12* CpG2, (%)	31.57 ± 4.61	35.70 ± 7.29	0.207	37.90 ± 7.56	37.66 ± 6.75	0.872

**Table 6:** Multiple linear regression between *P2Y12* methylation levels and clinical factors.

Model		*B*	*P*_coefficients_	*F*	*P*_*F* value_	*R* square
*P2Y12* CpG1 with alcohol abuse	(Constant)	33.069	0.012	9.302	0.001	0.724
	TBIL	−0.362	0.013			
	LEVF	0.690	0.001			
	Albumin	−0.755	0.019			
	AST	0.017	0.050			

*P2Y12* CpG2 with alcohol abuse	(Constant)	21.666	0.020	10.033	0.001	0.667
	LEVF	0.619	0.000^a^			
	Albumin	−0.668	0.010			
	AST	0.017	0.014			

*P2Y12* CpG1 with current smoking	(Constant)	28.035	0.004	6.193	0.005	0.251
	LEVF	0.318	0.026			
	TBIL	−0.314	0.049			

*P2Y12* CpG1 with Albumin <35	(Constant)	−19.001	0.109	14.345	0.000^a^	0.840
	LEVF	0.643	0.000^a^			
	CRP	0.129	0.001			
	BUN	2.228	0.006			
	Triglycerides	3.205	0.044			

^a^The *P* value is less than 0.001.

**Table 7:** Logistic regression analysis with nongenetic and genetic factors in total population.

Variables	*B*	*P* value	Exp (*B*)	95% C.I.
*P2Y12* CpG1	−0.141	0.096	0.869	0.74–1.03
*P2Y12* CpG2	0.169	0.142	1.184	0.95–1.49
Age	0.047	0.163	1.048	0.98–1.12
Gender (male)	−0.591	0.399	0.554	0.14–2.19
Hypertension	0.569	0.324	1.767	0.57–5.47
DM	−0.979	0.282	0.376	0.06–2.24
Dyslipidemia	−0.538	0.395	0.584	0.17–2.02
Current smoking	−0.378	0.573	0.685	0.18–2.55
Alcohol abuse	1.010	0.214	2.746	0.56–13.51
BMI	−0.073	0.514	0.930	0.75–1.16
Stent	0.865	**0.009 **	2.374	1.24–4.53
LEVF	−0.035	0.368	0.965	0.89–1.04
TC	0.093	0.847	1.097	0.43–2.82
Triglycerides	0.081	0.770	1.084	0.63–1.86
HDL	−1.283	0.308	0.277	0.02–3.27
LDL	−0.408	0.481	0.665	0.21–2.07
GLU	0.681	**0.013 **	1.976	1.15–3.39
HbAC1	−0.907	**0.022 **	0.404	0.19–0.88
ALT	−0.009	0.541	0.992	0.97–1.02
AST	0.002	0.366	1.002	1.00–1.01
TBIL	−0.036	0.357	0.965	0.89–1.04
A	−0.091	0.264	0.913	0.78–1.07
BUN	−0.161	0.292	0.851	0.63–1.15
CR	−0.004	0.825	0.996	0.97–1.03
UA	0.002	0.455	1.002	1.00–1.01
CRP	−0.007	0.677	0.993	0.96–1.03
PLT	0.008	0.712	1.008	0.97–1.05
MPV	0.380	0.507	1.462	0.48–4.50
PCT	−13.635	0.639	0.000	0.00–6.71*E* + 18
PDW	−0.183	0.736	0.832	0.29–2.42
Constant	8.480	0.469	4815.697	

**Table 8:** Logistic regression analysis with nongenetic and genetic factors in subgroup of alcohol abuse.

Variables	*B*	*P* value	Exp (*B*)	95% C.I.
*P2Y12* CpG 1	−0.362	0.242	0.697	0.38–1.28
*P2Y12* CpG 2	0.087	0.841	1.091	0.47–2.55
Current smoking	−0.512	0.845	0.599	0.00–101.18
Albumin	−0.046	0.799	0.955	0.67–1.36
Stent	−0.549	0.567	0.578	0.09–3.77
GLU	−0.212	0.884	0.809	0.05–13.84
HbAC1	−0.134	0.943	0.875	0.02–34.27
Constant	17.144	0.134	2.789*E* + 07	

**Table 9:** Logistic regression analysis with nongenetic and genetic factors in subgroup of current smoking.

Variables	*B*	*P* value	Exp (*B*)	95% C.I.
*P2Y12* CpG 1	−0.107	0.056	0.898	0.81–1.00
Alcohol abuse	0.291	0.707	1.338	0.29–6.12
Albumin	−0.088	0.241	0.916	0.79–1.06
Stent	0.430	0.328	1.537	0.65–3.64
GLU	0.577	0.150	1.781	0.81–3.91
HbAC1	−0.847	0.128	0.429	0.14–1.28
Constant	8.942	0.039	7648.274	

**Table 10:** Logistic regression analysis with nongenetic and genetic factors in subgroup of Albumin < 35.

Variables	*B*	*P* value	Exp (*B*)	95% C.I.
*P2Y12* CpG 1	−0.414	0.199	0.661	0.35–1.24
Current smoking	−9.623	0.922	0.000	0.00–2.95*E* + 79
Alcohol abuse	4.505	0.965	90.429	0.00–9.00*E* + 89
Stent	0.803	0.711	2.233	0.03–157.51
GLU	−2.065	0.340	0.127	0.00–8.79
HbAC1	1.789	0.573	5.986	0.01–3041.06
Albumin	−1.016	0.307	0.362	0.05–2.55
Constant	49.113	0.265	2.136*E* + 21	
